# Effect of Tofogliflozin on Skeletal Muscle Mitochondrial Function in Male Diabetic Mice With Muscle Atrophy

**DOI:** 10.1210/jendso/bvaf171

**Published:** 2025-11-03

**Authors:** Chiaki Kishida, Maki Murakoshi, Hiroko Sakuma, Terumi Shibata, Yusuke Suzuki, Tomohito Gohda

**Affiliations:** Department of Nephrology, Juntendo University Faculty of Medicine, Bunkyo-ku, Tokyo 113-8421, Japan; Department of Nephrology, Juntendo University Faculty of Medicine, Bunkyo-ku, Tokyo 113-8421, Japan; Department of Nephrology, Juntendo University Faculty of Medicine, Bunkyo-ku, Tokyo 113-8421, Japan; Department of Nephrology, Juntendo University Faculty of Medicine, Bunkyo-ku, Tokyo 113-8421, Japan; Department of Nephrology, Juntendo University Faculty of Medicine, Bunkyo-ku, Tokyo 113-8421, Japan; Department of Nephrology, Juntendo University Faculty of Medicine, Bunkyo-ku, Tokyo 113-8421, Japan

**Keywords:** AMP-activated protein kinase, exercise endurance, mitochondrial dynamics, sarcopenia, sodium-glucose cotransporter-2 inhibitor

## Abstract

Sodium-glucose cotransporter-2 (SGLT2) inhibitors are effective medications for type 2 diabetes (T2D), chronic kidney disease, and chronic heart failure regardless of diabetic status. However, concerns remain about their potential to reduce skeletal muscle mass. This study clearly demonstrates that tofogliflozin (Tofo), an SGLT2 inhibitor, improves skeletal muscle mitochondrial function, morphology, and performance in a mouse model of T2D with dexamethasone (Dex)-induced muscle atrophy. Obese diabetic KK-*A^y^* mice and nondiabetic KK mice were used. Muscle atrophy was induced in the KK-*A^y^* mice by intraperitoneal Dex injections for 2 weeks, followed by Tofo administration (0.015%) in the diet for 2 weeks. Tofo treatment enhanced exercise endurance, restored mitochondrial morphology, increased succinate dehydrogenase activity, and elevated protein expression of optic atrophy 1 and dynamin-related protein 1. These changes were associated with AMPK (adenosine monophosphate–activated protein kinase) activation and reduced expression of the mitokine growth differentiation factor-15. Although Tofo increased muscle cross-sectional area, it did not significantly affect overall body or muscle mass, nor grip strength, suggesting a preferential effect on slow-twitch oxidative fibers. Importantly, these benefits occurred without weight loss, likely due to maintained or increased food intake. These findings suggest that Tofo specifically ameliorates mitochondrial dysfunction and improves muscle quality and endurance in diabetic sarcopenia, especially under preserved nutritional conditions. Because Tofo is a highly selective SGLT2 inhibitor with distinct pharmacokinetic properties, these results are specific to Tofo and should not be generalized to all SGLT2 inhibitors. Further studies are warranted to determine whether similar effects are observed with other agents in this class.

Sarcopenia is associated with a reduction in activities of daily living, and higher risks of falls and fractures, frailty, hospitalization, and mortality, all of which underscore the need for targeted treatments for muscle atrophy [1-3]. The prevalence of sarcopenia is high in people with diabetes, and numerous bidirectional links exist between type 2 diabetes (T2D) and sarcopenia, such that the presence of one condition may increase the risk of developing the other [4].

Sodium-glucose cotransporter-2 (SGLT2) inhibitors are effective treatments for diabetes, chronic heart failure, and chronic kidney disease [5-7]. They are thought to increase glucagon secretion as a compensatory mechanism, thereby stimulating gluconeogenesis and lipolysis and reducing fat mass [8]. However, concerns exist that the activation of gluconeogenesis by SGLT2 inhibitors may promote muscle degradation and impair muscle protein biosynthesis, thereby contributing to sarcopenia [9, 10]. This concern has led to limitations in the use of SGLT2 inhibitors, particularly in older individuals with sarcopenia. However, studies performed in individuals with body mass indexes above the normal range have shown that these drugs reduce body and fat mass without reducing skeletal muscle mass, and that any reduction in skeletal muscle mass is relatively small, compared with the corresponding decrease in fat mass [11-14].

Mitochondrial function is often impaired in diabetes, and improvements in mitochondrial biogenesis and function have been shown to be associated with the alleviation of diabetic complications [15, 16]. SGLT2 inhibitors have been shown to improve mitochondrial function and biosynthesis and mitophagy in the heart and kidneys [17, 18]. Skeletal muscle mitochondrial dysfunction develops with age, decreases in physical activity, and chronic disease, resulting in lower adenosine triphosphate production, impaired bioenergetics, less mitochondrial biosynthesis, and greater oxidative stress and reactive oxygen species production, leading to muscle atrophy. Therefore, mitochondrial dysfunction is considered to be an important contributor to the development of skeletal muscle atrophy [19]. To model the compounded catabolic stress often seen in clinical settings, we administered dexamethasone (Dex) in addition to T2D, thus creating a more pathophysiologically relevant model of diabetic sarcopenia [20, 21]. However, few studies have focused on the mechanisms of the effects of SGLT2 inhibitors on skeletal muscle. Therefore, in the present study, we aimed to test the hypothesis that SGLT2 inhibitor treatment ameliorates sarcopenia by enhancing skeletal muscle mitochondrial function and improving muscle quality and endurance in a T2D mouse model with Dex-induced muscle atrophy.

## Materials and Methods

### Animals

Male KK-*A^y^*/Ta Jcl (a model of obese T2D) and KK/Ta Jcl (nondiabetic control) mice were purchased from CLEA Japan. Only male mice were used in this study to minimize potential confounding effects of sex hormones on muscle metabolism and mitochondrial function. The mice were individually housed in plastic cages with free access to a pelleted diet (Ce-2, CLEA Japan) and water throughout the experiment. The mice were housed in the same room under specific pathogen-free conditions and a 12-hour light/dark cycle, with the temperature controlled at 24 ± 1 °C. The animal experiments were approved by the ethics review committee for animal experimentation of the Juntendo University Faculty of Medicine (document No. 1568), and all the animals were treated according to the Guidelines for Animal Experimentation of Juntendo University, Tokyo, Japan. Twelve-week-old KK and KK-*A^y^* mice were randomly allocated to 4 groups: KK mice (nondiabetic control [non–DM-CTRL]), KK-*A^y^* mice (diabetic control [DM-CTRL]), Dex-treated KK-*A^y^* mice (diabetic with Dex treatment [DM-Dex]), and Dex- and tofogliflozin (Tofo)–treated KK-*A^y^* mice (diabetic with Dex and Tofo treatment [DM-Dex/Tofo]). Starting at age 12 weeks, all mice received daily treatments for 2 weeks. Mice in the non–DM-CTRL and DM-CTRL groups were administered daily intraperitoneal injections of saline. Mice in the DM-Dex and DM-Dex/Tofo groups received daily intraperitoneal injections of dexamethasone sodium phosphate (Dex, 20 mg/kg; 046-30813, Fujifilm Wako Pure Chemical Corporation) dissolved in saline. In addition, the DM-Dex/Tofo group was provided a diet containing 0.015% Tofo during the same 2-week period. This treatment protocol was designed to induce moderate muscle atrophy in diabetic KK-*A^y^* mice, enabling the evaluation of Tofo's effects while avoiding excessive muscle catabolism. Tofo was chosen because of its higher selectivity for SGLT2 over SGLT1 relative to other inhibitors, thereby minimizing off-target effects [22]. Additionally, its established clinical use in Japan enhances the translational relevance of our findings [23]. Tofogliflozin was generously provided by Kowa Company, Ltd. The grip strength and exercise endurance of the mice were measured at age 14 weeks. At the end of the experiment, blood samples were collected from the tail vein under anesthesia induced by intraperitoneal injection of pentobarbital (Somnopentyl, 65 mg/kg; Kyoritsu Seiyaku Corporation). The mice were then euthanized and perfused via the left ventricle with cooled 0.9% saline, and their kidneys, gastrocnemius muscles, soleus muscles, and quadriceps femoris muscles were collected.

### Physiological and Biochemical Measurements

Body mass, food intake, water intake, and glycated hemoglobin (HbA_1c_) levels of the mice were measured at ages 12 and 14 weeks. The blood HbA_1c_ levels were analyzed using a DCA 2000 HbA_1c_ immunoassay cassette and a DCA Vantage Analyzer (Siemens Healthcare).

### Measurement of Exercise Capacity

Forelimb grip strength was assessed using a grip force meter (MK-380Si; Muromachi Kikai). For each trial, mice were placed on the device and pulled parallel to the ground until they lost their grip. At each time point, the mice underwent 5 trials, with 10-second rests between these. A mean value was calculated for the data collected during the best 3 of the 5 trials, which was recorded as the final score for each mouse.

Exercise endurance was assessed using a treadmill (Exer 3/6; Columbus Instruments). The mice were placed on the treadmill and spent 10 minutes at rest, then they ran at a starting speed of 6 m/min for 10 minutes, and subsequently they ran at a speed that was increased at the rate of 2 m/min every 2 minutes until exhaustion. The treadmill gradient was maintained at 10%. The time to exhaustion was recorded as the time taken for the mice to stop running and remain on the electric shock grid for 10 seconds, without attempting to resume running.

### Muscle Histology

Gastrocnemius muscles were fixed in 4% paraformaldehyde, embedded in paraffin, sectioned, and stained with hematoxylin and eosin. The stained sections were imaged using a DP73 microscope (Olympus). Fifty muscle fibers from each mouse were randomly selected, and their cross-sectional areas (CSAs) were measured using ImageJ software (National Institutes of Health). The number of mice analyzed was 5 for the non–DM-CTRL group and 6 for the other groups.

### Immunohistochemical Analysis

To evaluate the succinate dehydrogenase (SDH) activities of gastrocnemius muscles, frozen sections were incubated at 37 °C for 45 minutes in a medium containing nitroblue tetrazolium (1.2 M), sodium succinate (0.2 M), and sodium phosphate buffer pH 7.5 (0.2 M). Images were acquired using the DP73 microscope. The immunohistochemical reactivity of 60 muscle fibers per mouse was then assessed using ImageJ software, with 4 mice analyzed for each experimental group.

### Transmission Electron Microscopy

For transmission electron microscopy (TEM), soleus muscles were fixed in 2% glutaraldehyde in 0.1-M phosphate buffer (pH 7.4) and postfixed in 1% OsO_4_ in the same buffer. The fixed specimens were dehydrated in ethanol and embedded in epoxy resin. Then, ultrathin sections were prepared and stained with uranyl acetate and lead citrate. The specimens were examined using a JEM-1400 microscope (JEOL). To quantitatively evaluate mitochondrial swelling, we analyzed two morphological parameters: area and circularity. Mitochondrial swelling is typically characterized by an increase in size and a more rounded morphology under stress conditions [24]. In this study, mitochondria with an area greater than the 95th percentile of the size distribution in the non–DM-CTRL group were defined as enlarged, representing outliers. A circularity value of 0.8 or greater was used to indicate a rounded morphology. Although a universally accepted definition is lacking, circularity of 0.8 or greater is commonly used as an empirical threshold in morphological analyses of cells and organelles. Therefore, this threshold was adopted based on its consistency with visual assessment and practical utility. Mitochondria that met both criteria (area >95th percentile and circularity ≥0.8) were defined as “swollen.” The proportion of swollen mitochondria was calculated and compared across groups. Area and circularity measurements were performed using ImageJ software based on the TEM images.

### Western Blot Analysis

Lysates were prepared from gastrocnemius muscles in RIPA buffer (radioimmunoprecipitation assay; Thermo Fisher Scientific) containing protease and phosphatase inhibitors (PhosSTOP phosphatase inhibitor; Roche). Electrophoresis, membrane transfer, and blocking were then performed sequentially. Membranes were incubated sequentially with primary and secondary antibodies. The primary antibodies used were rabbit anti-AMPKα (adenosine monophosphate [AMP]-activated protein kinase α, AMPKα; Cell Signaling, No. 2532; RRID: AB_330331), rabbit anti-phospho-AMPKα (Thr172) (Cell Signaling, No. 2531; RRID: AB_330330), rabbit anti-Akt (Protein kinase B, Akt; Cell Signaling, No. 4691; RRID: AB_915783), rabbit anti-phospho-Akt (Ser473) (Cell Signaling, No. 9271; RRID: AB_329825), rabbit anti-S6 (ribosomal protein S6, S6; Cell Signaling, No. 2217; RRID: AB_331355), rabbit anti-phospho-S6 (Ser235/236) (Cell Signaling, No. 2211; RRID: AB_331679), rabbit anti-MuRF1/2/3 (muscle RING finger proteins 1-3, MuRF1/2/3; Abcam, ab172479; RRID: AB_3712411), rabbit anti-myostatin (myostatin, MSTN; Abcam, ab71808; RRID: AB_1268982), rabbit anti-SIRT1 (sirtuin 1, SIRT1; SirT1 (D1D7) Rabbit mAb, Cell Signaling, No. 9475; RRID: AB_2617130), rabbit anti-PGC1α (peroxisome proliferator-activated receptor γ coactivator 1-α, PGC1α; Anti-PGC1α-N-terminus, Abcam, ab191838; RRID: AB_2721267), rabbit anti-OPA1 (optic atrophy 1, OPA1; Cell Signaling, No. 80471; RRID: AB_2734117), rabbit anti-DRP1 (dynamin-related protein 1, DRP1; Cell Signaling, No. 8570; RRID: AB_10950498), rabbit anti-GDF15 (growth differentiation factor-15, GDF15; Abcam, ab105738; RRID: AB_10937795), and mouse anti-GAPDH (glyceraldehyde-3-phosphate dehydrogenase, GAPDH; Abcam, ab9484; RRID: AB_307274). After enhanced chemiluminescence detection, the bands were visualized and analyzed using FusionCapt Advance Solo 7 software (Vilber-Lourmat).

### Statistical Analysis

Data normality was assessed using the Shapiro-Wilk test. Continuous data with a normal distribution were expressed as mean ± SD and compared using one-way analysis of variance, followed by the Bonferroni post hoc test. Categorical datasets were compared using the χ^2^ test or Fisher exact test. *P* less than .05 was considered to indicate statistical significance. All analyses were performed using EZR software (version 1.42, Saitama Medical Center, Jichi Medical University).

## Results

### Physiological and Biochemical Parameters in Mice Aged 12 and 14 Weeks

**Figure 1.**
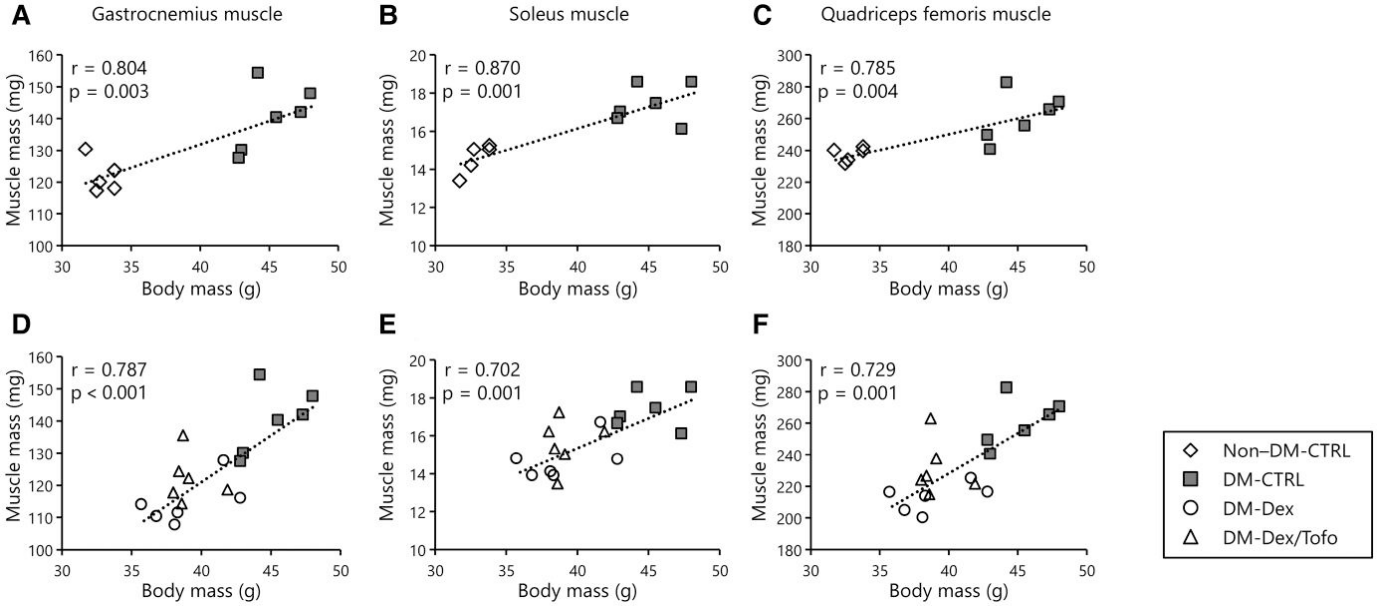
The relationship between body and muscle mass. A, Gastrocnemius muscle; B, soleus muscle; and C, quadriceps femoris muscle masses of the non–DM-CTRL and DM-CTRL groups. D, Gastrocnemius muscle; E, soleus muscle; and F, quadriceps femoris muscle masses of the DM-CTRL, DM-Dex, and DM-Dex/Tofo groups. Significant positive correlations between body and muscle mass were obtained for all groups. Abbreviations: DM-CTRL, KK-*A^y^* mice; DM-Dex, dexamethasone-treated KK-*A^y^* mice; DM-Dex/Tofo, dexamethasone and tofogliflozin-treated KK-*A^y^* mice; non–DM-CTRL, KK mice.

Before the experiment, we measured the baseline body weight (BW) and HbA_1c_ of each mouse at age 12 weeks, and confirmed that there were no significant differences between the DM-CTRL, DM-Dex, and DM-Dex/Tofo groups. As shown in Table 1, the masses of the gastrocnemius, soleus, and quadriceps femoris muscles were significantly lower in the DM-Dex group than in the DM-CTRL group, indicating the successful establishment of a muscle atrophy model induced by Dex. At age 14 weeks, both the body masses and skeletal muscle masses of the DM-Dex and DM-Dex/Tofo groups were significantly lower than those of the DM-CTRL group. Muscle mass in diabetic mice correlated more with BW than glucose control, and DM-CTRL mice exhibited higher muscle mass than nondiabetic controls (Fig. 1A-1F). The HbA_1c_ levels of the DM-Dex/Tofo group were significantly lower than those of the DM-CTRL and DM-Dex groups. Although the DM-Dex/Tofo group tended to have higher food intake than the DM-Dex group, this difference was not statistically significant. However, the water intake of the DM-Dex/Tofo group was significantly higher than that of the other groups. There were no significant differences in the grip strengths of the mice in any of the groups. The running distance and workload, assessed on the treadmill, were significantly lower in the DM-Dex group than in the DM-CTRL group, but these parameters were significantly higher in the DM-Dex/Tofo group.

**Table 1.** Biochemical parameters for each mouse group at ages 12 and 14 weeks

	Non–DM- CTRL	DM- CTRL	DM- Dex	DM- Dex/Tofo	*P*
No.	5	6	6	6	
Age 12 wk					
Body mass, g	32.3 ± 0.9	44.9 ± 2.0*^a^*	43.2 ± 2.0*^a^*	44.0 ± 0.9*^a^*	<.001
Glycated hemoglobin, %	4.2 ± 0.1	6.4 ± 1.1*^a^*	7.1 ± 0.8*^a^*	6.8 ± 1.0*^a^*	<.001
Age 14 wk					
Body mass, g	32.9 ± 0.8	45.2 ± 2.0*^a^*	39.0 ± 2.5*^a,b^*	39.2 ± 1.3*^a,b^*	<.001
Glycated hemoglobin, %	3.9 ± 0.4	7.5 ± 0.6*^a^*	8.3 ± 0.8*^a^*	5.7 ± 1.0*^a,b,c^*	<.001
Food intake, g/d	5.0 ± 0.1	5.8 ± 1.0	6.1 ± 0.8	7.3 ± 0.7*^a,b^*	<.005
Water intake, mL/d	6.5 ± 0.6	11.8 ± 1.6	16.9 ± 2.5*^a^*	26.0 ± 5.9*^a,b,c^*	<.001
Grip strength, g	231 ± 13	238 ± 20	235 ± 20	242 ± 17	.82
Running distance, m	760 ± 135	567 ± 119	345 ± 110*^a,b^*	634 ± 121*^c^*	<.001
Work, J	43.1 ± 7.5	43.8 ± 10.5	23.2 ± 8.5*^a,b^*	42.1 ± 7.1*^c^*	<.005
Muscle masses					
Gastrocnemius muscle, mg	122 ± 5	140 ± 9*^a^*	114 ± 6*^b^*	122 ± 7*^b^*	<.001
Soleus muscle, mg	14.6 ± 0.7	17.3 ± 0.9*^a^*	14.6 ± 1.0*^a^*	15.5 ± 1.2*^b^*	<.001
Quadriceps femoris muscle, mg	238 ± 4	260 ± 14*^a^*	212 ± 8*^a,b^*	230 ± 16*^b^*	<.001

Data are presented as mean ± SD.

Abbreviations: DM-CTRL, KK-*A^y^* mice; DM-Dex, dexamethasone-treated KK-*A^y^* mice; DM-Dex/Tofo, dexamethasone and tofogliflozin-treated KK-*A^y^* mice; non–DM-CTRL, KK mice.

^*a*^ *P* less than .05 vs non–DM-CTRL.

^*b*^ *P* less than .05 vs DM-CTRL.

^*c*^ *P* less than .05 vs DM-Dex.

### Effects of Tofogliflozin on the Kidneys

Consistent with previous reports [25, 26], Tofo exerted renoprotective effects in our model. Glomerular changes and mesangial expansion observed in the DM-CTRL and DM-Dex groups were ameliorated by Tofo, along with reduced expression of renal inflammation- and fibrosis-related genes (Supplementary Fig. S1A-S1H [27]).

### Mouse Cross-Sectional Areas of Gastrocnemius Muscles

**Figure 2.**
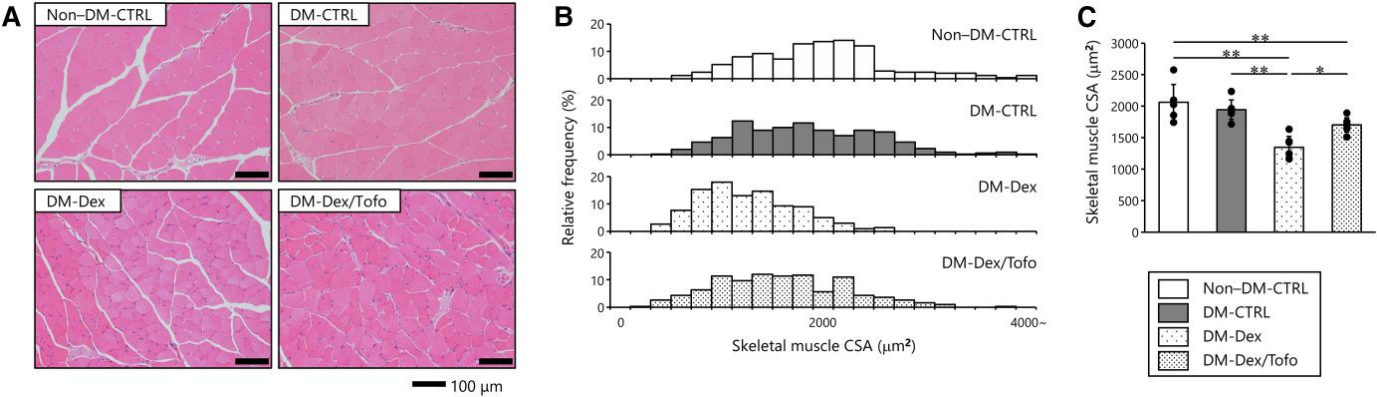
Morphology of the gastrocnemius muscles. A, Representative hematoxylin and eosin–stained gastrocnemius muscle sections (×200, scale bar: 100 µm). Histograms representing B, size distribution of skeletal muscle fibers and C, quantitative analysis of fiber size (n = 5 for the non–DM-CTRL group and n = 6 for the other groups, with 50 muscle fibers randomly selected for each mouse). Data are presented as mean ± SD. Tofogliflozin treatment significantly increased the skeletal muscle fiber size of mice treated with dexamethasone. Comparisons of groups were performed using one-way analysis of variance. *P less than .05; **P less than .01. Abbreviations used in this figure are the same as in Fig. 1. Abbreviation: CSA, cross-sectional area.

Fig. 2A shows representative light micrographs of gastrocnemius muscles from mice in each group. The histogram distribution of the skeletal muscle CSAs of the DM-Dex group showed a shift toward smaller CSAs vs the non–DM-CTRL and DM-CTRL groups. However, Tofo treatment shifted the distribution closer to that of the non–DM-CTRL and DM-CTRL groups (Fig. 2B). The CSAs of the DM-Dex group were significantly smaller than those of the non–DM-CTRL and DM-CTRL groups. Tofo treatment significantly increased these values compared with the DM-Dex group; however, they remained significantly smaller than those of the non–DM-CTRL group (Fig. 2C).

### Effects of Tofogliflozin on Protein Synthesis and Proteolysis Pathways in Gastrocnemius Muscle

**Figure 3.**
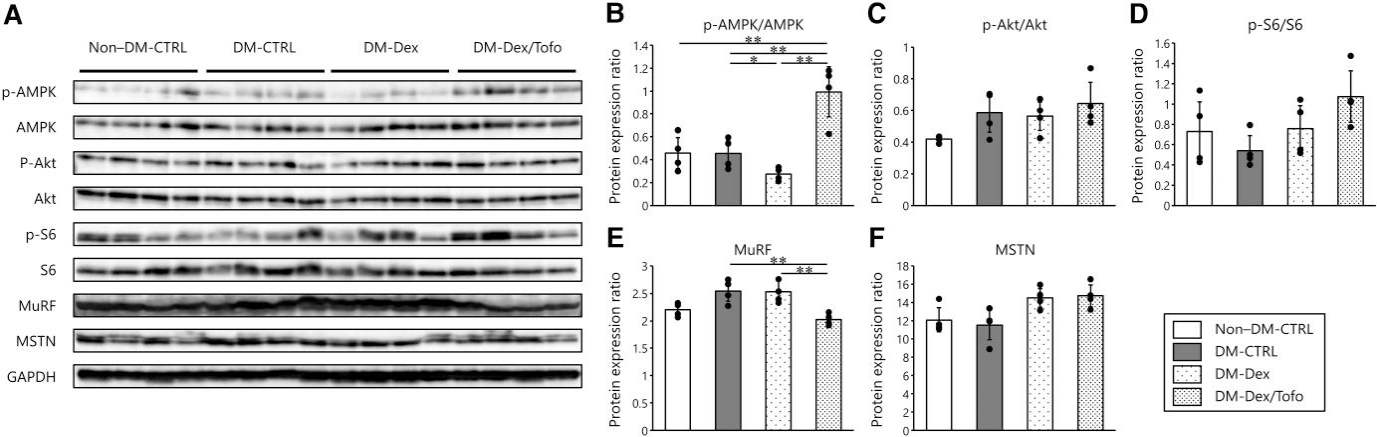
Expression of proteins involved in protein synthesis and proteolysis pathways in the gastrocnemius muscle. A, Western blot analysis of the protein expression of B, *P*-AMPK/AMPK; C, *p*-Akt/Akt; D, *p*-S6/S6; E, MuRF; and F, MSTN in the gastrocnemius muscle. Tofogliflozin treatment significantly increased the activity of AMPK. However, there were no differences between the groups in the activation of proteins related to the other protein synthesis pathways or in MSTN expression. Tofogliflozin treatment significantly reduced MuRF expression vs the other diabetic groups. Comparisons of groups were performed using one-way analysis of variance. *P less than .05; **P less than .01. Abbreviations used in this figure are the same as in Fig. 1. Abbreviations: Akt, protein kinase B; AMPK, adenosine monophosphate–activated protein kinase; GAPDH, glyceraldehyde 3-phosphate dehydrogenase; MSTN, myostatin; MuRF, muscle RING-finger protein; S6, ribosomal protein S6.

Fig. 3A shows the protein expression levels for the gastrocnemius muscles of mice in each group. To determine whether Tofo treatment affected skeletal muscle atrophy and muscle synthesis, we measured the expression of proteins associated with catabolism and anabolism in these muscles. The level of AMPK activation in the DM-Dex/Tofo group was significantly higher than that in the other groups (Fig. 3B). However, there were no differences in the activities of Akt or S6, which are involved in the mTOR (mechanistic target of rapamycin) signaling pathway of protein synthesis (Fig. 3C and 3D). The expression of MuRF, a ubiquitin ligase, was significantly lower in the DM-Dex/Tofo group than in the DM-CTRL and DM-Dex groups (Fig. 3E), but there were no significant differences in the expression of myostatin, a muscle growth inhibitor (Fig. 3F). Thus, overall, Tofo had minimal effects on protein synthesis and proteolysis pathways in skeletal muscle.

### Amelioration of Abnormalities in Mitochondrial Morphology in Soleus Muscle and Impaired Mitochondrial Activity in Gastrocnemius Muscle by Tofogliflozin

**Figure 4.**
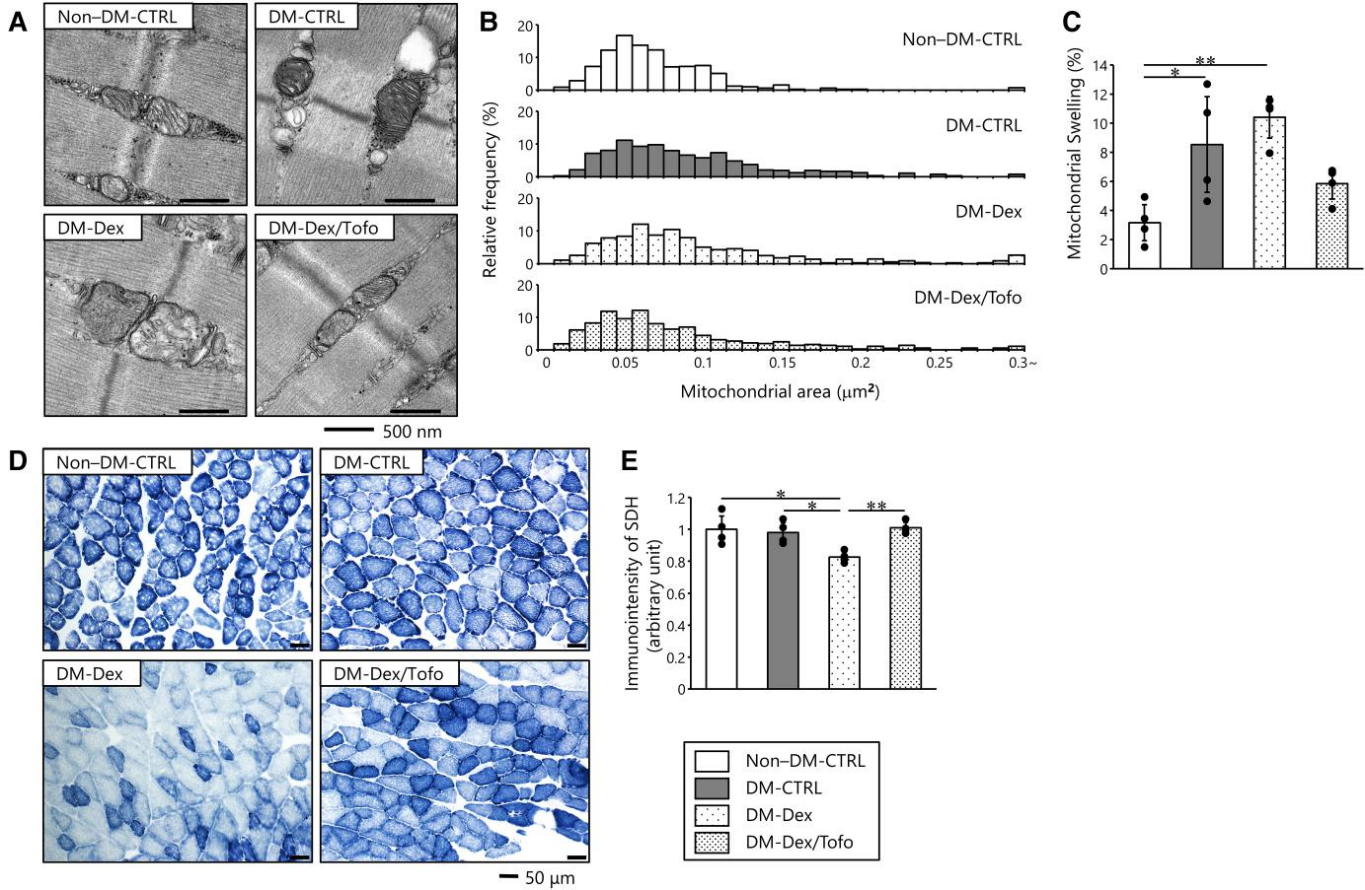
Amelioration of the abnormalities in mitochondrial morphology in soleus muscle and the impaired mitochondrial activity in gastrocnemius muscle by tofogliflozin. A, Representative transmission electron microscopy images of soleus muscle sections (×20,000, scale bar: 500 nm). B, Histogram showing the distribution of mitochondrial area in soleus muscle. The DM-CTRL and DM-Dex groups showed a shift toward larger mitochondrial areas compared to the non–DM-CTRL group, with the DM-Dex group showing the most pronounced shift. Tofogliflozin treatment restored the distribution closer to that of the non–DM-CTRL group. C, Quantitative analysis of mitochondrial swelling in soleus muscle. Swollen mitochondria were defined as having an area greater than the 95th percentile of the non–DM-CTRL group and a circularity of 0.8 or greater. The proportion of swollen mitochondria was significantly higher in the DM-CTRL and DM-Dex groups than in the non–DM-CTRL group. The DM-Dex/Tofo group showed a trend toward reduction in swelling compared to the DM-Dex group (*P* = .062). D, Representative gastrocnemius muscle sections stained for SDH (×200, scale bar: 20 µm). E, Quantitative analysis of the SDH activity (n = 4 per group, with 60 muscle fibers randomly selected from each mouse). Abnormalities in mitochondrial morphology, such as bulky shape and disorganized cristae, and lower SDH staining intensity, were identified after dexamethasone treatment, and these were ameliorated by tofogliflozin treatment. Data are presented as mean ± SD. Comparisons of groups were performed using one-way analysis of variance. *P less than .05; **P less than .01. Abbreviations used in this figure are the same as in Fig. 1. Abbreviation: SDH, succinate dehydrogenase.

The ultrastructural morphology of the soleus muscle was analyzed using TEM. The DM-Dex group was characterized by destruction and swelling of the mitochondrial cristae, but these defects were ameliorated by Tofo treatment (Fig. 4A). The histogram distribution of mitochondrial showed a rightward shift both in the DM-CTRL and the DM-Dex groups compared to the non–DM-CTRL group, with the shift being most prominent in the DM-Dex group. Tofo treatment appeared to restore the distribution toward that observed in the non–DM-CTRL group (Fig. 4B). The proportion of swollen mitochondria was significantly higher in the DM-CTRL and DM-Dex groups than in the non–DM-CTRL group. Although the difference was not statistically significant, the proportion tended to be lower in the DM-Dex/Tofo group than in the DM-Dex group (*P* = .062) (Fig. 4C). Representative cross-sections of gastrocnemius muscles stained for SDH activity, a marker of mitochondrial respiratory chain complex II, are shown in Fig. 4D. The SDH staining was less intense in the DM-Dex group than in the DM-CTRL group, and this defect was also ameliorated by Tofo treatment (Fig. 4E).

### Effects of Tofogliflozin on Mitochondrial Biogenesis, Dynamics, and Stress

**Figure 5.**
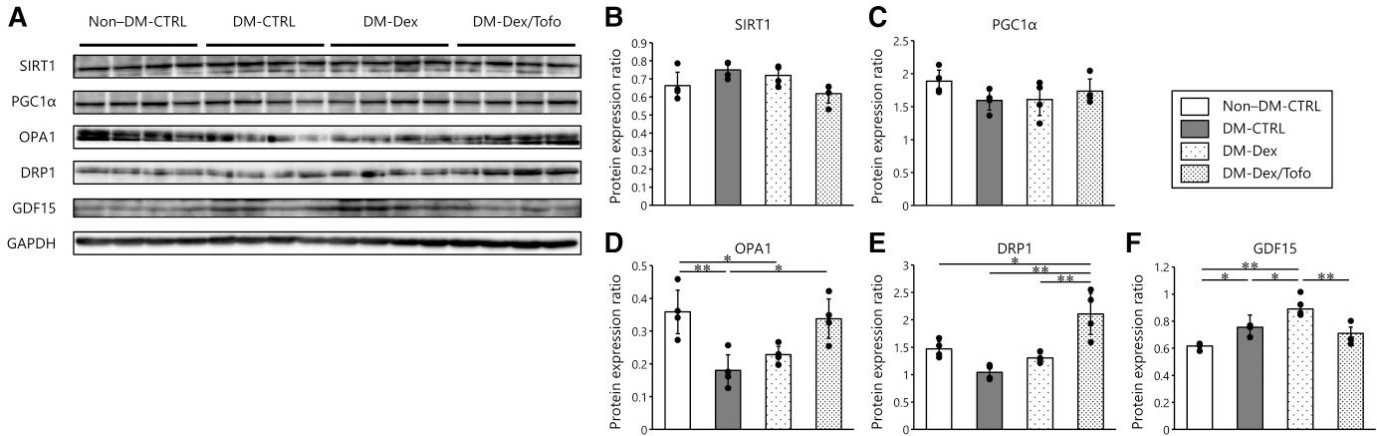
Expression of proteins involved in mitochondrial function in the soleus muscle. A, Western blot analysis of the protein expression of B, SIRT1; C, PGC1α; D, OPA1; E, DRP1; and F, GDF15 in the soleus muscles. No significant differences were identified for SIRT1 and PGC1α expression. DM significantly reduced OPA1 expression, and this was restored by tofogliflozin treatment. In addition, tofogliflozin treatment significantly increased DRP1 expression vs the other groups. Tofogliflozin treatment significantly reduced GDF15 expression vs the other DM groups. Comparisons of groups were performed using one-way analysis of variance. *P less than .05; **P less than .01. Abbreviations used in this figure are the same as in Fig. 1. Abbreviations: DRP1, dynamin-related protein 1; GAPDH, glyceraldehyde 3-phosphate dehydrogenase; GDF15, growth differentiation factor-15; OPA1, optic atrophy 1; PGC1α, peroxisome proliferator–activated receptor γ coactivator 1-α; SIRT1, sirtuin 1.

We next evaluated the effects of Tofo on mitochondrial biogenesis, dynamics, and stress. Representative immunoblots are shown in Fig. 5A. We measured the expression of SIRT1 and PGC1α, which are involved in the AMPK/SIRT1/PGC1α pathways and regulate mitochondrial biogenesis, and found no differences in the expression of SIRT1 or PGC1α among the groups (Fig. 5B and 5C). To determine the effects of the drug on mitochondrial dynamics and autophagy/mitophagy, we measured the expression of OPA1 and DRP1, proteins that are associated with mitochondrial fusion and fission, in the soleus muscle. OPA1 expression was significantly higher in the DM-Dex/Tofo group than in the other groups (Fig. 5D). DRP1 expression was significantly reduced by Dex treatment, but Tofo treatment significantly attenuated this effect (Fig. 5E). To characterize mitochondrial stress, we measured the expression of GDF15, a known mitokine, in the soleus muscle. This was significantly lower in the DM-Dex/Tofo group than in the DM-CTRL and DM-Dex groups (Fig. 5F).

## Discussion

In the present study, we have demonstrated for the first time that the SGLT2 inhibitor Tofo improves the mitochondrial function, muscle fiber size, and exercise endurance of mice with a combination of obesity, T2D, and glucocorticoid-induced muscle atrophy, without inducing weight loss. These beneficial effects appear to be mediated, at least in part, by AMPK activation and the restoration of mitochondrial dynamics.

The mouse model of a combination of diabetes with Dex-induced stress closely recapitulates the catabolic state that is often seen clinically, in which coexisting conditions exacerbate muscle wasting. The utility of this approach is evidenced by the small muscle CSA of the DM-Dex mice, which exhibited more pronounced atrophy than mice with diabetes alone. Although we did not identify robust activation of catabolic pathways that are typically activated by Dex [20, 21], the model recapitulated key features of muscle atrophy, including fiber size reductions and functional impairment. This apparent discrepancy might be explained by the contribution of Dex-induced hyperglycemia to the overall catabolic state or by transient changes in signaling at time points at which this was not assessed [28, 29].

Although the DM-CTRL mice had greater absolute muscle mass than the non–DM-CTRL group, this is likely because of their higher BW, which is a feature of the KK-*A^y^* diabetic model. This finding is consistent with previous findings that individuals with T2D often have greater body mass, which is associated with higher absolute muscle mass without correspondingly superior muscle quality or function [30]. However, in the present study, the muscle fibers of the diabetic mice had small CSAs, implying poor muscle quality. This contrast underscores the limitations of relying solely on total muscle mass as an indicator of muscle health, because it may reflect noncontractile tissue accumulation or be confounded by overall body size [31]. In comparison, the CSA is a more specific index of myofiber morphology and functional integrity.

Tofo treatment increased the muscle CSA and endurance of the mice without corresponding effects on grip strength or total muscle mass. There are several possible explanations for this discrepancy. First, grip strength primarily reflects the instantaneous force of forearm muscles, which may not directly correspond to the force generated by the hindlimb muscles in which fiber CSA was measured. Second, muscle CSA provides a more specific measure of myofiber hypertrophy, suggesting that the increase induced by Tofo represents a genuine morphological adaptation, which may be an early structural change that precedes overt hypertrophy [32, 33]. Third, fiber type–specific effects may be involved: SGLT2 inhibitors are known to preferentially enhance mitochondrial and metabolic function in slow-twitch fibers [34], which are critical for endurance. Finally, the improvement in exercise capacity may also reflect the well-established cardiovascular benefits of SGLT2 inhibitors, such as the improvement in cardiac function, which would also improve endurance [6, 35].

We found that Tofo treatment increased mitochondrial enzyme activity, as demonstrated by an increase in SDH staining, and also partially restored DRP1 expression and increased AMPK phosphorylation. Low expression of mitochondrial fusion/fission regulators such as OPA1 and DRP1 is a known feature of obesity-induced diabetes [36, 37], and the fact that Dex did not further reduce OPA1 or DRP1 expression suggests that these pathways were already significantly dysregulated in the diabetic state. The present finding that Tofo-induced AMPK activation is linked to improvements in mitochondrial dynamics is consistent with previous research that showed that OPA1 is crucial for mitochondrial fusion and respiratory efficiency [38] and that its expression can be restored via AMPK activation [39]. Although the results of SDH staining provide strong evidence of an improvement in mitochondrial activity, future studies using complementary methods such as high-resolution respirometry are necessary to provide a more comprehensive assessment. AMPK activation was identified in the absence of concurrent changes in mTOR/S6 signaling, suggesting the involvement of alternative pathways, consistent with the findings of other studies of SGLT2 inhibitors [40, 41]. These findings indicate that the beneficial effects observed with Tofo may extend beyond glucose lowering and involve diverse AMPK-mediated mechanisms.

Meta-analyses have shown slight reductions in lean mass in association with the use of SGLT2 inhibitors, and this is often confounded by concurrent weight loss and variable nutritional status [42-44]. Consistent with findings made in well-nourished patients [11-14], the present model showed the preservation of muscle mass and superior function. These results underscore the importance of considering nutritional status when evaluating the muscle-related effects of SGLT2 inhibitors.

The present study had several limitations. First, we did not include a group receiving Tofo alone (without Dex) or a comparison with other antidiabetic agents. This study was designed specifically to examine the effects of Tofo under Dex-induced catabolic stress, and therefore additional treatment arms were beyond the scope and available resources. Future studies should include these additional groups to clarify whether the observed muscle-protective effects are unique to Tofo or shared by other glucose-lowering interventions. Second, we cannot rule out the possibility that the observed effects were partly related to improvements in glycemic control [45]. Third, the findings cannot be fully generalizable to other SGLT2 inhibitors as this study focused solely on Tofo, which is not a prototype of the SGLT2 inhibitor class but rather a highly selective agent with distinct pharmacological characteristics. Therefore, the observed effects should be interpreted as drug-specific, and further studies are required to clarify whether similar benefits occur with other SGLT2 inhibitors. Fourth, the relatively short duration of Tofo treatment in the study limits our ability to evaluate its long-term effects. Therefore, future studies involving longer treatment periods are warranted to confirm the durability of the identified improvements in mitochondrial function and muscle performance. Finally, we studied only male mice, and potential sex differences remain to be explored.

In conclusion, the present study was the first to demonstrate that Tofo improves mitochondrial dysfunction, muscle fiber atrophy, and endurance in a mouse model of obesity, T2D, and glucocorticoid-induced muscle loss. These beneficial effects, which were likely mediated by an AMPK-dependent restoration of mitochondrial dynamics, occurred without reductions in body or muscle mass. The findings highlight the therapeutic potential for Tofo with respect to muscle atrophy in patients with metabolic diseases. However, given that Tofo is not a prototype representative of the SGLT2 inhibitor class, these results should be interpreted as drug-specific and not generalized to other SGLT2 inhibitors. Further studies comparing multiple SGLT2 inhibitors are needed to clarify whether similar benefits occur across this class.

## Data Availability

Data will be made available on reasonable request.

## Abbreviations

AMPK: adenosine monophosphate–activated protein kinase
BW: body weight
CSA: cross-sectional area
Dex: dexamethasone
DM-CTRL: diabetic control
DM-Dex: diabetic with dexamethasone treatment
DM-Dex/Tofo: diabetic with dexamethasone and tofogliflozin treatment
DRP1: dynamin-related protein 1
HbA_1c_: glycated hemoglobin
mTOR: mechanistic target of rapamycin
non–DM-CTRL: nondiabetic control
OPA1: optic atrophy 1
SDH: succinate dehydrogenase
SGLT2: sodium-glucose cotransporter-2
T2D: type 2 diabetes
TEM: transmission electron microscopy
Tofo: tofogliflozin
